# Der Einsatz problembasierten Lernens im Blended-Learning-Studienmodell zur Erhöhung der Lernaktivität

**DOI:** 10.1365/s40702-021-00795-z

**Published:** 2021-09-27

**Authors:** Alina Bockshecker, Katharina Ebner, Stefan Smolnik, Christian Anschütz

**Affiliations:** Lehrestuhl Betriebswirtschaftslehre, insbes. Betriebliche Anwendungssysteme, FernUniversität in Hagen, Hagen, Deutschland

**Keywords:** Blended Learning, Digitales Lehr‑/Lernkonzept, Gamification, Gestaltungsanforderungen, Lernerfolg, Problembasiertes Lernen, Blended Learning, Design Requirements, Digital Education Concept, Gamification, Learning Success, Problem Based Learning

## Abstract

Die digitale Transformation mit ihrer Komplexität und Vielfältigkeit stellt Lehrende vor große Herausforderungen, geeignete Lehr‑/Lernkonzepte zu entwickeln. Zusätzlich ergeben sich durch Blended-Learning-Studienmodelle weitere Herausforderungen in Bezug auf die didaktische und organisatorische Umsetzung von Lehr‑/Lernkonzepten. Ziel dieses Beitrags ist es, ein Lehr‑/Lernkonzept vorzustellen, das die digitale Transformation sowohl inhaltlich als auch didaktisch auf Basis von problembasiertem Lernen, Gamification und virtueller Gruppenarbeit umsetzt. In der Gruppenarbeit setzen sich die Studierenden inhaltlich mit vielfältigen Konzepten der digitalen Transformation auseinander und werden darauf vorbereitet, die immer neuen und komplexen Aufgaben der digitalen Transformation bewältigen zu können. Es werden zudem die notwendigen Gestaltungsanforderungen dargelegt und die zugrundeliegenden Gestaltungszyklen kurz beschrieben.

## Abstract (en)

The digital transformation with its complexity and diversity presents lecturers with major challenges in developing an education concept for this. In addition, blended learning study models pose further challenges with regard to the didactic and organizational implementation of such education concepts. The aim of this paper is to present a concept that implements the digital transformation both in terms of content and didactics based on problem-based learning, gamification and virtual group work. In group work, students deal with the content of diverse complex concepts of digital transformation and they are also prepared to be able to master new and complex tasks of digital transformation. The necessary design requirements are also briefly outlined and the underlying design cycle is briefly described.

## Einleitung

Die Gestaltung und Integration innovativer digitaler Lehr‑/Lernkonzepte haben nicht zuletzt im Kontext der Covid-19-Schutzmaßnahmen deutlich an Relevanz gewonnen und werden nachhaltig von vielen Hochschulen forciert. Die Digitalisierung der Lehre „muss [dabei] zu einem besseren Lernen und damit zu grundlegenden Verbesserungen der Hochschulbildung beitragen“ (Rachel 2019) und sollte nicht dem Selbstzweck der Digitalisierung dienen. In der wissenschaftlichen aber auch praxisorientierten Literatur zur Digitalisierung der Lehre werden verschiedene Lehr‑/Lernkonzepte diskutiert (z. B. der Einsatz von Virtual Reality, Elmqaddem 2019). Herausforderungen derartiger Lehr‑/Lernkonzepte umfassen einerseits die didaktische Umsetzung einer mit der Präsenzlehre vergleichbar intensiven und qualitativen Wissensvermittlung (Freeman und Urbaczewski 2019) sowie die Sicherstellung von Lern- bzw. Studienpersistenz und nachhaltigem Lern- bzw. Studienerfolg (Xu und Jaggars 2013). Andererseits stellt die digitale Transformation in ihrer Komplexität und Vielfalt Lehrende vor Herausforderungen, geeignete Lehr‑/Lernkonzepte inhaltlich zu gestalten, da nicht nur die relevanten technischen und technologischen Innovationen zu betrachten sind. Studierende müssen auch den Einfluss der digitalen Transformation auf nahezu alle Lebensbereiche (Hess 2019, S. 18) aus soziotechnischer Perspektive begreifen. Komplexe Veränderungen der Beziehungen zwischen Mensch, Aufgabe und Technik bilden die Basis einer Vielzahl von teilweise widersprüchlichen Konzepten, die verstanden werden müssen. Dies ist jedoch in Gänze nur schwer in einem einzigen Modul umsetzbar.

Ziel dieses Beitrags ist es daher, die didaktische Gestaltung, Realisierung und Evaluation eines Lehr‑/Lernkonzepts der FernUniversität in Hagen vorzustellen, das inhaltlich in die Begriffe, Konzepte und Modelle der digitalen Transformation einführt. Durch die Implementierung einer virtuellen Gruppenarbeit in Kombination mit einem problembasierten, gamifizierten Lernansatz zu Problemen aus dem Bereich „Digitalisierung von Städten und Verkehr“ im Bachelor-Modul „Digitale Transformation“ der Fakultät für Wirtschaftswissenschaft an der FernUniversität in Hagen werden die Studierenden darauf vorbereitet, die immer neuen und komplexen Aufgaben der digitalen Transformation bewältigen zu können.

Das beschriebene Lehr‑/Lernkonzept ist auf Fernlehre ausgerichtet, d. h. auf eine minimale physische Präsenz sowie Lernen auf Distanz. Die zugrundeliegenden didaktischen Konzepte – problembasiertes Lernen, virtuelle Gruppenarbeit und Gamification – sowie deren Integration in das Lehr‑/Lernkonzept zur Adressierung der oben skizzierten Herausforderungen werden vorgestellt. Darüber hinaus gibt der Beitrag einen Ausblick, wie das Lehr‑/Lernkonzept weiterentwickelt und auf andere Module übertragen werden kann.

### Problembasiertes Lernen als Konzept zur Vermittlung von Inhalten der digitalen Transformation

Die digitale Transformation bezeichnet den durch Informations- und Kommunikationstechnologien (IKT) hervorgerufenen Wandel (Hess 2019) von Individuen, Unternehmen und ganzen Gesellschaften. Menschen, Aufgaben und Technik werden weiter vernetzt, neue Technologien eingesetzt sowie größere Datenmengen gesammelt, die mit entsprechender Analytik ausgewertet werden. In diesem Zusammenhang werden oft die SMAC-Technologien (**s**oziale Medien, **m**obile IKT, fortschrittliche (Daten-)**A**nalytik sowie **C**loud-Computing) angeführt, die zu vielfältigen Veränderungen – auch in Lehr‑/Lernkonzepten – beitragen (Khanm 2020). Die digitale Transformation stellt mit ihrer Komplexität und Vielfältigkeit Lehrende vor Herausforderungen, Studierende auf zukünftige Problemsituationen im Arbeitsleben vorzubereiten. Aus diesem Grund ist es sinnvoll, den Studierenden zwar die Grundlagen der digitalen Transformation zu vermitteln, sie aber vielmehr zu befähigen, selbstständig die Problemsituationen zu bearbeiten und geeignete Lösungen zu entwickeln. Darum fokussiert das im weiteren Verlauf vorgestellte Lehr‑/Lernkonzept auf die didaktische Umsetzung in Form einer virtuellen Gruppenarbeit.

Problembasierte Lehr‑/Lernkonzepte beruhen auf selbstgesteuerten Lernprozessen anhand von komplexen, unstrukturierten, oft widersprüchlichen und offenen Problemsituationen sowohl alleine als auch in der Gruppe (Müller Werder 2013). Die Lernenden sollen dabei anhand einer gegebenen Problemsituation selbstständig relevante Lerninhalte identifizieren, eigene Wissenslücken feststellen und schließen sowie eine mögliche Lösung erarbeiten. Diese Lösung ist dabei nicht a priori festgelegt, sondern von den individuellen Interpretationen und dem Wissensstand der Lernenden abhängig. Problembasiertes Lernen ermöglicht es, umfassendes und transferfähiges Wissen zu erwerben sowie effektive Problemlösekompetenzen zu entwickeln. Ein zentraler Unterschied zu traditionellen Lehr‑/Lernkonzepten liegt darin, dass das erworbene Wissen nicht bei der Problembearbeitung abgefragt wird, sondern im Zuge dieser erst entsteht (Müller Werder 2013). Die Problemsituation erzeugt kognitive Konflikte und Interaktionen zwischen den Lernenden, die den Stimulus für das Lernen darstellen und die Motivation sowie die Lernbereitschaft fördern. Darüber hinaus wird die langfristige kognitive Verankerung der gelernten Inhalte, eine Erweiterung der Problemlösungsfähigkeiten sowie das Denken höherer Ordnung und der Erwerb von Fähigkeiten zum selbstgesteuerten und lebenslangen Lernen gefördert (Hung et al. 2008).

**Abb. 1:**
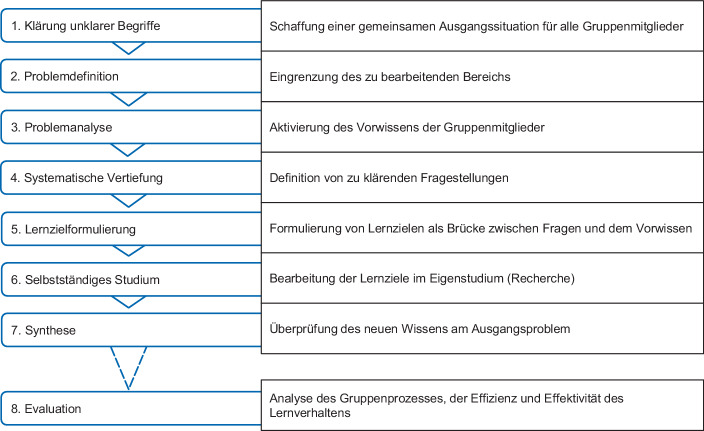
Schritte des problembasierten Lehr‑/Lernkonzepts in der virtuellen Gruppenarbeit. (In Anlehnung an Müller Werder 2013; Schmidt 1983)

Der Siebenschritt (Abb. 1, engl. 7‑step) stellt hierbei die didaktische Prozessstruktur des problembasierten Lernens dar (Schmidt 1983; Müller Werder 2013); sie dient den Studierenden als Orientierung sowie Strukturierung der problembasierten Gruppenarbeit. Die Evaluation der eigenen Lösung und Herangehensweise in der Gruppe stellt einen achten Schritt dar, der von der thematisch-inhaltlichen Problembearbeitung in der Gruppe losgelöst ist. Der Evaluation kommt als Reflexionsschritt im Rahmen des problembasierten Lernens große Bedeutung zu, da die Lernenden hierbei ihre eigenen Lernstrategien, den Gruppenprozess, die erlernten Inhalte und erarbeitete Lösung kritisch bewerten sowie dabei metakognitiv verarbeiten, was zentral für die Lernpersistenz ist (Xu und Jaggars 2013).

### Gestaltunganforderungen und zyklische (Weiter‑)Entwicklung des Lehr‑/Lernkonzepts

Die Gestaltung des hier vorgestellten Lehr‑/Lernkonzepts erfolgte anhand identifizierter Gestaltungsanforderungen (GAen) in insgesamt drei Gestaltungszyklen. Bedingt durch das Blended-Learning-Studienmodell der FernUniversität in Hagen, das aus einer Kombination von Online- und Präsenzlehre besteht, ist das Modul auf Fernlehre ausgerichtet, d. h. auf eine minimale physische Anwesenheit der Studierenden sowie Lernen auf Distanz. Die Studierenden absolvieren ihr Studium oft neben einer Berufstätigkeit in Voll- oder Teilzeit, sodass Flexibilität und Optionalität der Gruppenarbeit wichtige Kriterien für den Erfolg des Lehr‑/Lernkonzepts sind. Zudem sind die Studierenden über den gesamten deutschsprachigen Raum, einige sogar international, verteilt. Das Modul und damit einhergehend die Gruppenarbeit müssen mit den zur Verfügung stehenden Ressourcen umsetzbar sein, d. h. große Studierendenzahlen sind, in dem vorliegenden Fall, mit zwei wissenschaftlichen Mitarbeiterinnen und einer studentischen Hilfskraft zu organisieren und umzusetzen. Um nachhaltigen Studien- bzw. Lernerfolg insbesondere in einem Fernstudium zu erreichen, ist eine weitere Anforderung, das Lehr‑/Lernkonzept motivierend zu gestalten, z. B. durch die Integration von Spielelementen („Gamification“), die die inhaltliche als auch organisatorische Auseinandersetzung fördern. Hieraus ergeben sich die fünf GAen:

- *GA1* (Virtualität): Physische Treffen der Studierenden sind zur Bearbeitung der Gruppenarbeit optional zu halten.
- *GA2* (Flexibilität): Räumliche und zeitliche Flexibilität der Gruppenarbeit muss gegeben sein.
- *GA3* (Optionalität): Das Modul muss auch ohne die Gruppenarbeit erfolgreich abzuschließen sein, d. h. Studierende können sich bewusst gegen eine Teilnahme an der Gruppenarbeit entscheiden.
- *GA4a* (prüfungsrechtliche Umsetzbarkeit): Die prüfungsrechtlichen Rahmenbedingungen müssen eingehalten werden und Bestandteile der Gruppenarbeit klar definiert sein.
- *GA4b *(Umsetzbarkeit mit verfügbaren Lehrstuhlressourcen): Das Konzept muss mit den zur Verfügung stehenden Ressourcen umsetzbar sein, d. h. auch bei sehr großen Belegerzahlen skalieren.
- *GA5* (Engagement und Leistungsbereitschaft): Der Studienerfolg soll sichergestellt werden durch Gamification-Ansätze.

Auf Basis des ADDIE-Prozesses (engl. Abkürzung für Analyse, Design, Entwicklung, Implementierung und Evaluation) nach Branch (2009) erfolgte die zyklische Entwicklung des virtuellen und auf dem Konzept des problembasierten Lernens basierenden Lehr‑/Lernkonzepts für das Modul „Digitale Transformationen“. Die GAen prägen die einzelnen Gestaltungszyklen.

Die erste Phase des ADDIE-Prozesses fokussiert hierbei auf die Analyse der Modulziele (Vermittlung der Basis sowie die Befähigung, sich selbstständig in neue Problemsituationen im Kontext der digitalen Transformation einzuarbeiten), der Zielgruppe (Studierende der Betriebs- und Volkswirtschaftslehre sowie der Wirtschaftsinformatik mittleren Alters und zu großen Teilen berufstätig) sowie der zur Verfügung stehenden Lehrstuhlressourcen für die Umsetzung des Konzepts (zunächst zur Verfügung stehende Konferenztechnik (Adobe Connect) und Lernmanagementsystem (Moodle) sowie zwei wissenschaftliche Mitarbeiterinnen und eine studentische Hilfskraft). In der Design-Phase sind die Modulstruktur mit klaren Verbindungen zwischen Inhalten und Lernzielen sowie Einführung in Inhalt und problembasiertes Lernen, die Lernziele für fünf thematische Moduleinheiten sowie die Prüfungsbestandteile und -leistungen zu definieren. In der darauffolgenden Phase der Entwicklung sind die Lernressourcen (z. B. Problemsituationen für die Gruppenarbeit, methodische Einführungen, Hinweismaterial) zu erstellen und die erforderlichen Ressourcen sowie Organisation der Gruppenarbeit konkreter auszugestalten. In der Implementierungsphase werden die neuen Lernressourcen eingesetzt und das erarbeitete Lehr‑/Lernkonzept umgesetzt. Die Betreuungspersonen unterstützen in dieser Phase die Studierenden als Lernbegleiter und Ansprechpersonen. In der Evaluationsphase wird die Zielerreichung geprüft und Feedback der Studierenden mit den formulierten Anforderungen abgeglichen; ggf. stellen diese als veränderte Anforderungen die Basis für den nächsten Gestaltungszyklus dar.

## Ausgestaltung eines virtuellen problembasierten Lehr‑/Lernkonzepts für die digitale Transformation

Das in drei ADDIE-Zyklen gestaltete Lehr‑/Lernkonzept für das Modul „Digitale Transformation“ wird in den folgenden Abschnitten näher dargestellt und erläutert.

### Modulstruktur und Prüfungsleistungen

Das Modul „Digitale Transformation“ ist in fünf thematische Einheiten^1^ mit je einem Arbeitsaufwand von 60 h gegliedert. Das problembasierte Lernen, dem Maastrichter Modell (siehe z. B. Müller Werder 2013) folgend, ist in der dritten Einheit in Form einer Gruppenarbeit integriert, um den Studierenden die oben genannten Fähigkeiten im Kontext der digitalen Transformation zu vermitteln. In der Gruppenarbeit können insgesamt 20 von 100 Modulpunkten erreicht werden. Die Gruppenarbeit ist in drei Teile gegliedert, wobei die bewertbaren Einzelleistungen die Gruppenleistung in Summe überwiegen:

1. Einzelleistung I: Inhaltliche Vorbereitung und Ausarbeitung jeweils einer Rolle und eines argumentativen Vortrags in Form eines 90-sekündigen Elevator Pitch (Denning und Dew 2012) (entspricht ca. einer halben Seite Fließtext).
2. Gruppenleistung: Gestaltung eines einseitigen Gruppenhandouts und von Folien für eine Abschlusspräsentation sowie Erarbeitung einer Lösung für die Problemsituation in Form eines 3‑minütigen Wrap-up.
3. Einzelleistung II: Erstellung einer individuellen maximal zweiseitigen schriftlichen Reflexion der Gruppenarbeit.

Mit dieser Ausgestaltung des problembasierten Lernens können verschiedene Anforderungen adressiert werden. Die Studierenden sind gefordert, spielerisch eine archetypische Rolle und die jeweiligen Argumentationen mit Konzepten der digitalen Transformation auszugestalten (GA5). Hierbei sind Abwägungen von einzelnen Argumenten zu treffen, inhaltliche Diskussionen mit den Kommilitonen zu führen sowie Lösungen zu debattieren. Darüber hinaus ermöglicht dieses Lehr‑/Lernkonzept gegenüber anderen Ausgestaltungsmöglichkeiten, auch große Teilnehmerzahlen zu betreuen und zu prüfen (GA4b) sowie eine virtuelle Umsetzung und Durchführung (GA1).

Die restlichen 80 Punkte entfallen auf eine Modulabschlussklausur. Die Teilnahme an der Gruppenarbeit ist für die Studierenden folglich insofern freiwillig, als dass das Modul auch ohne die Gruppenarbeit bestanden werden kann, allerdings können die 20 hierdurch zu erreichenden Punkte nicht kompensiert werden (GA3).

### Kontext der Problemsituationen

**Abb. 2:**
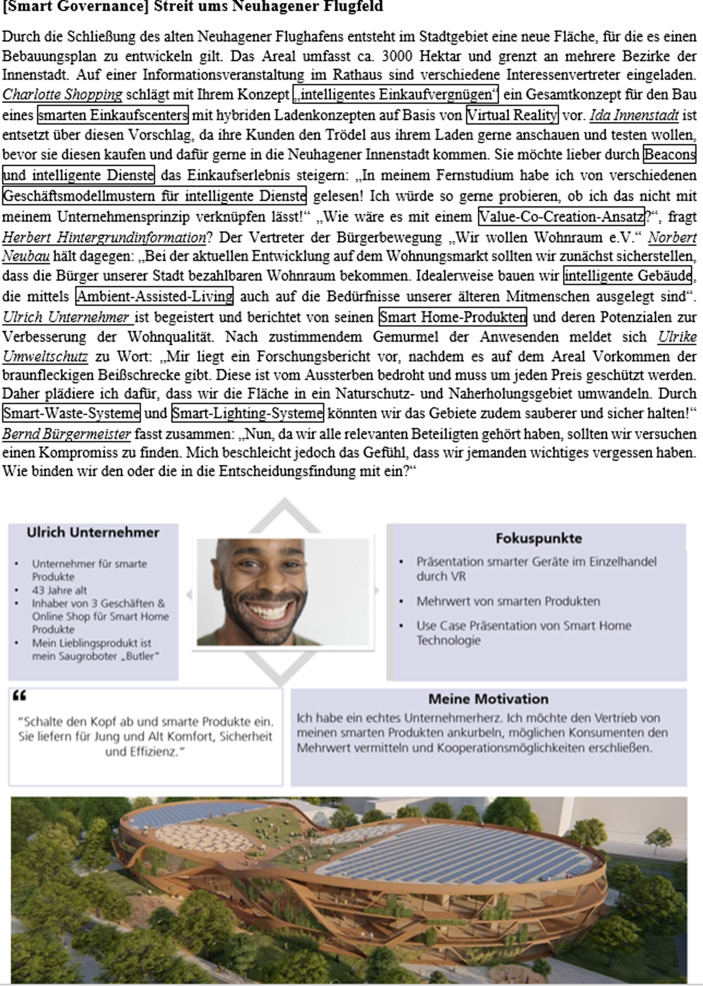
Exemplarische Problemsituation (*oben*, Konzepte der digitalen Transformation sind *eingerahmt* und die verschiedenen Rollen sind *unterstrichen*), Ausgestaltung einer Rolle (*Mitte*) und mögliche Lösungsidee (*unten*)

Im thematischen Kontext der Digitalisierung von Städten und Verkehr sind die soziotechnischen Verflechtungen der digitalen Transformation besonders ausgeprägt, weshalb Problemsituationen aus diesem Kontext für die Gruppenarbeit gewählt wurden. Basierend auf den sechs Smart-City-Domänen von Giffinger und Haindlmaier (2010) (Smart Economy, Smart People, Smart Governance, Smart Mobility, Smart Environment sowie Smart Living) werden komplexe Problemsituationen mit jeweils mindestens acht verschiedenen archetypischen Rollen des Stadtentwicklungskomitees der fiktiven Stadt Neuhagen (z. B. Bernd Bürgermeister, Cornelia Contra, Herbert Hintergrundwissen, Ulrich Unternehmer) entwickelt. Die Rollen vertreten verschiedene Positionen in Hinblick auf die jeweilige Problemsituation und gehen dabei auf widersprüchliche, komplexe und vielfältige Konzepte der digitalen Transformation ein. Insgesamt sind aktuell 15 Problemsituationen verfügbar. Die Problemsituationen orientieren sich hierbei an aktuellen technischen Entwicklungen und realen Problemen, wie sie in den letzten Jahren in Medien und (Fach‑)Presse diskutiert worden sind. Abb. 2 stellt eine beispielhafte Problemsituation sowie die Ausgestaltung einer Rolle dar.

Ein möglicher Lösungsansatz der Fallstudie (vgl. Abb. 2, unten) ist ein umfassendes Flächennutzungskonzept, in dem die verschiedenen Nutzungsideen der Fallstudie aufgegriffen und zusammengeführt werden.

### Ablauf und Umsetzung der Gruppenarbeitsphase

Die Studierenden werden zunächst im Rahmen einer Kick-off-Veranstaltung sowie anhand begleitender Materialien in das problembasierte Lernen sowie den Siebenschritt eingeführt. Hinweise zu den Bestandteilen der Gruppenarbeit bieten Unterstützung, um zum einen Unsicherheiten bzgl. des Prüfungsformats zu adressieren und zum anderen auch die spätere Bewertung der unterschiedlichen Bewertungsbestandteile zu konkretisieren (GA4a).

Um die studentischen Gruppen effizient einzuteilen, wird die Gruppeneinteilungsaktivität der Lernplattform (Moodle) verwendet. Hierbei können die Studierenden sich selbstständig in Gruppen von 5–8 Studierenden einteilen. In einem (Excel‑)Formular werden die Gruppenmitglieder, die Gruppensprecher/in für den Austausch mit dem Lehrstuhl sowie die Präferenzen bzgl. Problemwahl und Abschlusspräsentationstermin gesammelt und effizient erfasst (GA4b). Für die virtuelle Zusammenarbeit der Gruppen wird jeweils ein Jitsi-Raum zur Verfügung gestellt, um Diskussionen und den Austausch untereinander zu ermöglichen (GA1, GA2). Einmal in der Woche stehen die Betreuungspersonen in Form einer virtuellen Sprechstunde über Zoom für Fragen zur Verfügung. Zusätzlich besteht für alle Gruppen die Möglichkeit einer optionalen kurzen Zwischenpräsentation nach der Hälfte der Gruppenarbeitszeit von sechs Wochen, um den Fortschritt nach den ersten vier Schritten des Siebenschritts (vgl. Abb. 1) vorzustellen und Feedback zu erhalten sowie offene Fragen zu klären (GA2). Die Möglichkeit, Zwischenergebnisse zu präsentieren, ist notwendig, da die freie Arbeit mit den komplexen Problemsituationen schnell auch überfordernd wirken kann (Mansor et al. 2015), sollten Studierende mit diesen Lehr‑/Lernkonzepten nicht vertraut sein. Die Zwischen- und Abschlusspräsentationen finden auf der Zoom-Plattform statt (GA1), da sich diese als technisch stabiler im Vergleich zu Adobe Connect herausgestellt hat.

### Evaluation und weitere Schritte

Das Feedback der Studierenden zu der Umsetzung des problembasierten Lernens und der Lernzielerreichung am Ende des Wintersemesters 2019/2020 (erste Durchführung) war insgesamt positiv. Die Modulevaluation von 28 Studierenden zeigte zudem eine sehr gute Durchschnittsnote von 1,43 für das Modul (Bewertung von 1–5, von sehr gut bis mangelhaft). Die Studierenden wiesen jedoch auf einige zu verbessernde Aspekte hin: die technische Umsetzung der virtuellen Gruppenarbeit (Wechsel von Adobe Connect zu Jitsi und Zoom, GA1, GA2), die zeitliche Ausgestaltung der Gruppenarbeitsphase für Studierende (Ausweiterung der Gruppenarbeitsphase von vier auf sechs Wochen, GA2) sowie weitere verschriftlichte Erklärungen zur Gruppenarbeit und zum problembasierten Lernen (in Form von Hinweisen). Darüber hinaus wurde eine studentische Hilfskraft für die organisatorische Unterstützung in den Folgesemestern eingesetzt, die zum einen konkrete Erwartungshaltungen bzgl. der Zwischenpräsentationen kommuniziert und zum anderen die inhaltliche Ausgestaltung der Gruppenarbeiten ergänzt hat (vor allem Formulierung weiterer Problemsituationen und Rollen).

In den Folgesemestern gab es kaum konkrete neue Verbesserungsvorschläge seitens der Studierenden, allerdings ergaben sich durch die steigenden Teilnehmerzahlen an der virtuellen Gruppenarbeit (WS 2019/2020: 95 [6]^2^; SS 2020: 150 [3]; WS 2020/2021: 229 [14] sowie SS 2021: 208 [N/A] Studierende) Optimierungsbedarfe bei der Betreuung des Moduls. Die effiziente Gestaltung der Gruppenpräsentationen sowie die Betreuung großer Belegerzahlen stehen somit im Vordergrund der aktuellen Weiterentwicklungen. Es wird derzeit analysiert, wie die Gruppenarbeit noch effizienter ausgestaltet werden kann, welche Beratungsaspekte (weiter) automatisiert und spielerisch gestaltet werden können sowie an welcher Stelle eine individuelle Unterstützung der Studierenden erforderlich ist.

Mit einem ganzheitlichen Gamification-Konzept sollen darüber hinaus weitere Anreize geschaffen werden, dass die Studierenden ihren Lernprozess nicht nur während der Gruppenarbeitsphase, in der das problembasierte Lernen verankert ist, sondern während des Semesters aktiv mitgestalten (GA5). Hierfür ist es relevant, die unterschiedlichen Motive und Ziele der Studierenden in Bezug auf das erfolgreiche Abschließen des Moduls zu kennen und bei der Gestaltung dieser Gamification-Elemente bereits im Vorfeld zu berücksichtigen. Ein Stufensystem mit bedingter Freischaltung soll die Studierenden bei der strukturierten und schrittweisen Durchführung der Gruppenarbeit anhand des Siebenschritts unterstützen. Nach dem vierten Schritt der systematischen Vertiefung bekommen die Studierenden z. B. Hilfestellungen zu der Formulierung von Lernzielen, die im darauffolgenden fünften Schritt zu erarbeiten sind. Die Freischaltung höherer Stufen erfolgt jeweils mit Motivationsbotschaften wie z. B. „Yeah, nun bist du bereit für die Zwischenpräsentation!“ oder „Herzlichen Glückwunsch. Du hast alle Schritte des problembasierten Lernens abgeschlossen und bist nun reif für die Abschlusspräsentation!“. Die bedingte Freischaltung unterstützt zudem die Betreuungspersonen insofern, als dass Fragen zu den weiteren Prozessschritten, aber auch vorbereitende Arbeiten der Studierenden stärker angeleitet und begleitet werden (z. B. für die Zwischenpräsentation). Mit Abzeichen wie z. B. „Bug Hunter“ für die Identifikation von Fehlern in den Kursmaterialien sollen die Studierenden zudem einen extrinsischen Anreiz bekommen, bei der Verbesserung der Modulunterlagen mitzuwirken. Schließlich sollen die Studierenden zukünftig die Gesichter ihrer Rollen als Avatare in Moodle einbinden können. Die verschiedenen Motive und Ziele der Studierenden sollen im finalen Konzept durch unterschiedliche Gamification-Elemente Berücksichtigung finden, sodass möglichst alle Studierenden entsprechend ihrer Präferenzen gamifizierte Unterstützung erhalten. So stellen z. B. zu erreichende Abzeichen für den Spielertyp „Achiever“ einen Anreiz zur aktiven Teilnahme an der gamifizierten Lernumgebung dar, während für „Explorer“ neue Entdeckungsmöglichen in Moodle größere Anreize schaffen. Das Gamification-Konzept befindet sich derzeit in der für eine erste Umsetzung vorbereitenden Planung.

### Übertragbarkeit des Ansatzes und Ausblick

Das vorgestellte Lehr‑/Lernkonzept stellt eine Ausgestaltungsmöglichkeit des problembasierten Lernens in virtueller Form dar und kann auf andere Lehrveranstaltungen übertragen sowie bei Bedarf angepasst werden. Andere Formen der Ausgestaltung des problembasierten Lernens sind z. B. bei Müller Werder (2013) zu finden. Insbesondere durch die aktuellen Covid-19-Schutzmaßnahmen ergeben sich durch das virtuelle Format auch für Lehr‑/Lernkonzepte in Präsenz Potenziale, da die einzelnen Schritte des problembasierten Lehr‑/Lernkonzepts eben nicht unbedingt in Person vor Ort durchlaufen werden müssen, sondern in ein virtuelles oder hybrides Format übertragen werden können.

Ein wie in diesem Beitrag erläutertes problembasiertes Lehr‑/Lernkonzept ist generell „kein didaktischer Selbstläufer“ (Müller Werder 2013, S. 70), sondern bedarf einer stetigen Unterstützung der Studierenden durch Betreuungspersonen. Dies ist insbesondere auch in einer virtuellen Umsetzung der Fall, da Studierenden dort oftmals der Austausch mit den Kommilitonen fehlt. In einem solchen Lehr‑/Lernkonzept ist aber insbesondere die Kommunikation und Interaktion für eine erfolgreiche Bearbeitung der Gruppenarbeit unerlässlich. Der organisatorische Aufwand der Gruppenarbeit ist bei großen Belegerzahlen zwar durch automatisierte Gruppenwahlaktivitäten sowie durch eine konsolidierte Informationsübermittlung der Gruppen an den Lehrstuhl reduzierbar. Dennoch bleibt der Gesamtaufwand insbesondere durch die Bewertung der einzelnen Prüfungsleistungen und Abstimmung mit den Betreuungspersonen bestehen. Auch durch „Probleme bei der kooperativen Zusammenarbeit in [den] Gruppen“ (Müller Werder 2013) sowie durch Abbruch der Gruppenarbeit bedarf es stellenweise der Intervention der Betreuungspersonen.

Durch die auf dem Konzept des problembasierten Lernens basierende und während der Vorlesungszeit laufende Gruppenarbeit erfolgt eine grundlegende Einarbeitung in die Themen des Moduls (dies wurde auch in der Modulevaluation durch die Studierenden bestätigt). Die Gruppenarbeit führt zudem zu einer hohen Teilnahmequote der Studierenden an der Modulabschlussklausur. Während sich in anderen Modulen oft viele Studierende kurz vor der Klausur noch abmelden, wirkt sich die Gruppenarbeit positiv auf die Klausurteilnahmequote aus. Die Differenz zwischen der Anzahl der Gruppenarbeits- und Klausurteilnehmer lag in den letzten drei Semestern bei jeweils 15–20 Studierenden. Allerdings liegt dies auch in der Tatsache begründet, dass es prüfungsrechtlich nicht möglich ist, die erreichten Punkte aus der Gruppenarbeit in Folgesemester zu übernehmen. Dies bedeutet, dass bei Nichtteilnahme an der Klausur die Gruppenarbeitspunkte verfallen und die Gruppenarbeit im Folgesemester wiederholt werden muss, um die gesamten Modulpunkte erreichen zu können. Die Aktivitätsrate, die das Verhältnis zwischen Belegerzahlen und Klausurzahlen veranschaulicht, ist mit Werten zwischen 35–50 % in den letzten vier Semestern höher als die durchschnittliche Aktivitätsrate an der Fakultät von ca. 30 %. Auch die Durchschnittsnote der Klausur liegt mit rund 2,1 über der Durchschnittsklausurnote anderer Module der Fakultät von ca. 2,6.

Insgesamt ist die Implementierung des virtuellen Lehr‑/Lernkonzepts basierend auf dem problembasierten Lernen eine Bereicherung des Fernlehrestudiums. Der rege Austausch der Studierenden untereinander und mit den Betreuungspersonen ist für die Weiterentwicklung des Lehr‑/Lernkonzepts wertvoll. Beispielsweise ergaben sich Impulse für die Durchführung von Gastvorträgen und die Aktualisierung von Modulinhalten. Ein weiterer Vorteil des Konzepts ist, dass die Modulinhalte nicht nur für die Prüfung gelernt werden, sondern substanzieller kognitiv verarbeitet werden und so über einen längeren Zeitraum präsent sind. Durch die komplexen Problemsituationen wird das gelernte Wissen in Alltagsituationen anwendbar (Müller Werder 2013). Weitere Forschung sollte dennoch der Frage nachgehen, wie sich die Übertragung eines solchen, auch für die Studierenden aufwendigeren, Lehr‑/Lernkonzepts auf andere Module und die Erfolge der Studierenden auswirkt, wenn mehrere Module parallel in einem Semester diese Konzepte einsetzen. Das beschriebene Lehr‑/Lernkonzept zum Modul „Digitale Transformation“ an der FernUniversität in Hagen sowie die Entwicklung entlang von Gestaltungszyklen unterstützen dabei, dass die digitale Transformation „zu grundlegenden Verbesserungen der Hochschulbildung“ (Rachel 2019) beiträgt.

## Footnotes

1 (1) Konzepte und Technologien der digitalen Transformation, (2) Veränderte Wertschöpfung durch die Digitalisierung, (3) Digitalisierung von Städten und Verkehr, (4) Digitalisierung der Finanzbranche, sowie (5) Nutzung und Erfolg von Informationssystemen im Zeitalter der Digitalisierung.

2 Anzahl Studierende, die an der Klausur, nicht aber an der virtuellen Gruppenarbeit teilgenommen haben.

## References

[CR1] Branch RM (2009) Instructional design: the ADDIE approach. Springer US, Boston

[CR2] Denning PJ, Dew N (2012) The myth of the elevator pitch. Commun ACM 55(6):38–40. 10.1145/2184319.2184333

[CR3] Elmqaddem N (2019) Augmented reality and virtual reality in education. Myth or reality? Int J Emerg Technol Learn 14(3):234–242. 10.3991/ijet.v14i03.9289

[CR4] Freeman L, Urbaczewski A (2019) Critical success factors for online education: longitudinal results on program satisfaction. CAIS 44:630–645. 10.17705/1CAIS.04430

[CR5] Giffinger R, Haindlmaier G (2010) Smart cities ranking: an effective instrument for the positioning of the cities? ACE Archit City Environ 4(12):7–26. 10.5821/ace.v4i12.2483

[CR6] Hess T (2019) Willkommen in der digitalen Unternehmenswelt. In: Thomas H (Hrsg) Digitale Transformation strategisch steuern: Vom Zufallstreffer zum systematischen Vorgehen. Springer, Wiesbaden, S 11–39

[CR7] Hung W, Jonassen DH, Liu R (2008) Problem-based learning. Handb Res Educ Commun Technol 3(1):485–506

[CR8] Khanm HU (2020) The role of SMAC (social media, mobility, analytics, cloud) for students and educators in online education. J Theor Appl Inf Technol 98(6):915–934

[CR9] Mansor AN, Abdullah NO, Abd Wahab J, Sattar Rasul M, Mohd Nor MY, Raof RA (2015) Managing problem-based learning: challenges and solutions for educational practice. Asian Soc Sci 11(4):259–268. 10.5539/ass.v11n4p259

[CR10] Müller Werder C (2013) Problem-based Learning erfolgreich gestalten. In: Bachmann H (Hrsg) Hochschullehre variantenreich gestalten. Ansätze, Methoden und Beispiele rund um Kompetenzorientierung. Forum Hochschuldidaktik und Erw. Bildung. hep, Bern, S 50–77

[CR11] Rachel T (2019) Die Digitalisierung der Bildung ist kein Selbstzweck. https://www.bmbf.de/de/die-digitalisierung-der-bildung-ist-kein-selbstzweck-9695.html. Zugegriffen: 17. Febr. 2021 (Bundesministerium für Bildung und Forschung)

[CR12] Schmidt HG (1983) Problem-based learning: rationale and description. Med Educ 17(1):11–16 6823214 10.1111/j.1365-2923.1983.tb01086.x

[CR13] Xu D, Jaggars SS (2013) The impact of online learning on students’ course outcomes. Evidence from a large community and technical college system. Econ Educ Rev 37:46–57. 10.1016/j.econedurev.2013.08.001

